# Supplementary material for: Silkworm Pupae Function as Efficient Producers of Recombinant Glycoproteins with Stable-Isotope Labeling
Source: Biomolecules. 2020 Oct 26;10(11):1482. doi: 10.3390/biom10111482 (PMC7692867; doi:10.3390/biom10111482)
Supplement: Supplementary file 1 [file biomolecules-10-01482-s001.pdf]

Figure S1

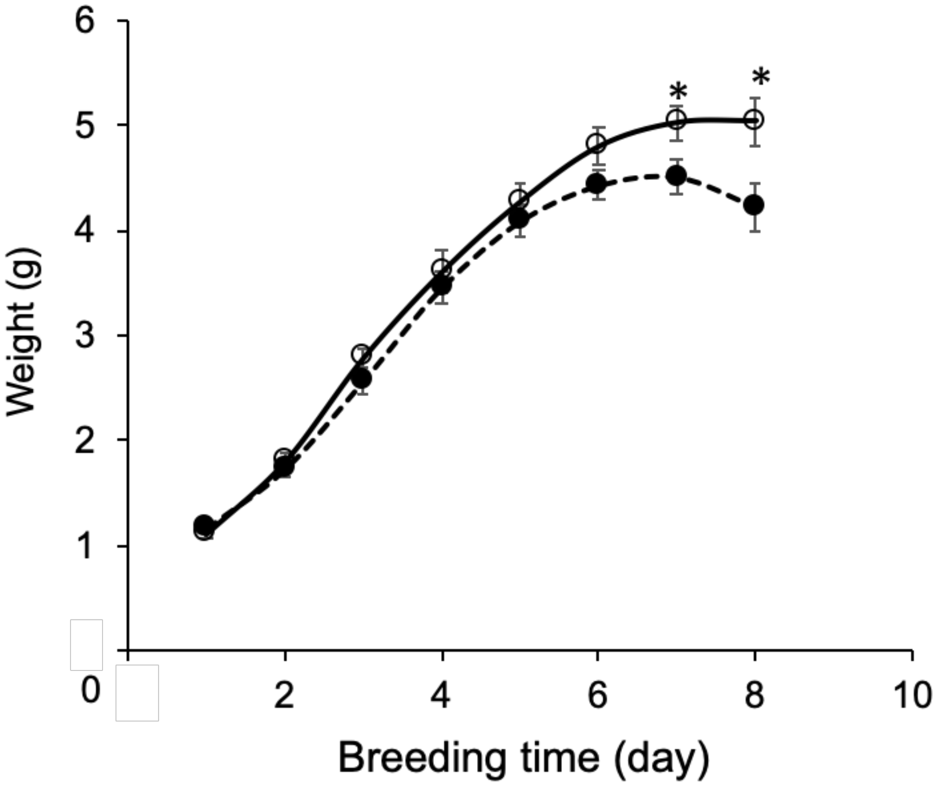

Figure S1: Effects of bacmid injection on silkworm larvae growth. Growth curve of silkworm larvae (n = 6) reared on artificial diet A20M20 with (closed circle) or without (open circle) bacmid injection on the second day. Means ± s.e.m. values are shown, **P*<0.0005 by Student's *t*-test.

Figure S2

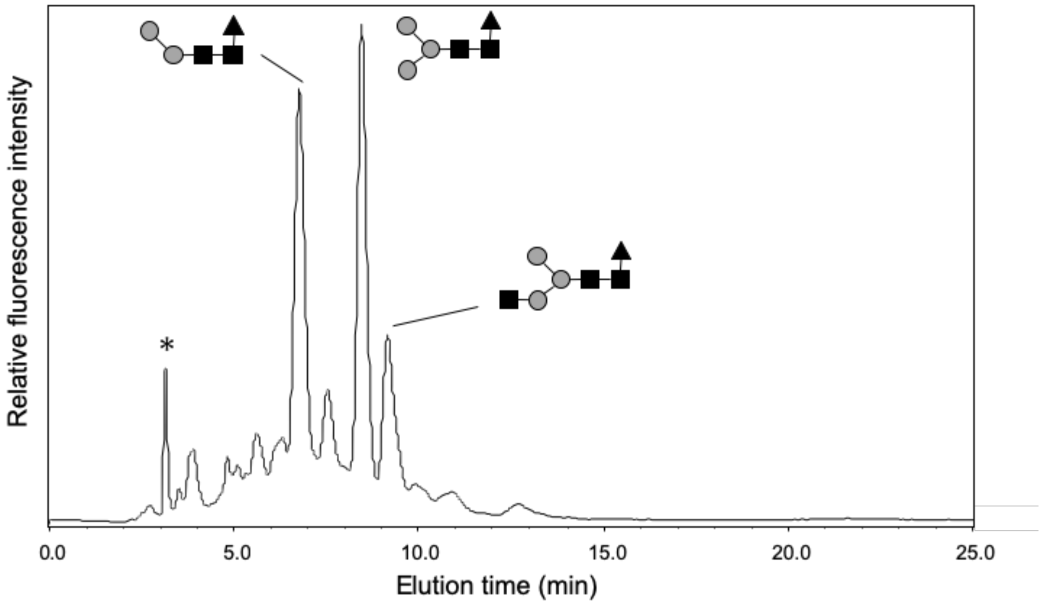

Figure S2: *N*-glycosylation profile of IgG expressed by silkworm pupae reared on artificial diet A20M20. Key symbols: Fuc (triangle), GlcNAc (square), Man (circle). Asterisk represents contaminating signal.
